# Constitutively overexpressed 21 kDa protein in Hodgkin lymphoma and aggressive non-Hodgkin lymphomas identified as cytochrome B_5b _(CYB5B)

**DOI:** 10.1186/1476-4598-9-14

**Published:** 2010-01-26

**Authors:** Derek Murphy, Jeremy Parker, Minglong Zhou, Faisal M Fadlelmola, Christian Steidl, Aly Karsan, Randy D Gascoyne, Hong Chen, Diponkar Banerjee

**Affiliations:** 1 Centre for Human Proteomics, Royal College of Surgeons in Ireland, 126 St Stephen's Green, Dublin 2, Ireland; 2 Centre for Translational and Applied Genomics, British Columbia Cancer Agency, Provincial Health Services Authority Laboratories, 600 West 10th Avenue, Vancouver, British Columbia, V5Z 4E6, Canada; 3 British Columbia Cancer Research Centre, 675 West 10th Avenue, Vancouver, British Columbia, V5Z 1L3, Canada; 4 University of Botswana, Biomedical Sciences, School of Medicine, Private Bag UB 0022, Gaborone, Botswana

## Abstract

**Background:**

We have previously reported a novel constitutively overexpressed 21 kDa protein in Hodgkin Lymphoma (HL) and aggressive Non-Hodgkin Lymphomas (NHL). The objective of the current study was to 1) identify this protein using two independent methods, 2) study the expression of the protein and its encoding mRNA in reactive lymph nodes, normal lymphocytes and CD34+ bone marrow precursor cells, 3) analyse patterns of expression of the protein in tissue microarrays assembled from a large number of diagnostic clinical biopsies from patients with HL, and 4) determine the copy number variation and mutation status of the encoding gene in HL cell lines.

**Results:**

Peptide sequencing by LC-MS/MS and protein identification by protein array screening identified a single protein, CYB5B. No mutations were detected in the *CYB5B *gene in HL cell lines. Quantitative PCR showed *CYB5B *gene expression was increased in HL and NHL cell lines. Array CGH using a submegabase resolution tiling array revealed gains in the *CYB5B *locus in HL cell lines KMH2 and L428. Membrane expression was seen in Reed-Sternberg cells in clinical biopsies from patients with HL but not in reactive lymph nodes. Bone marrow CD34+ precursor cells were CYB5B negative on the cell surface. RT-PCR assays of RNA extracted from T and B cell enriched fractions obtained from normal peripheral blood mononuclear cells, reactive lymph nodes, tonsils and normal bone marrow samples showed no evidence of increased mRNA levels of *CYB5B *in comparison to housekeeping gene *GAPDH*.

**Conclusions:**

The 21 kDa protein overexpressed in HL and aggressive NHL is identical to CYB5B. *CYB5B *gene expression is increased in a subset of HL and NHL cell lines tested. This is associated with *CYB5B *gene amplification in HL cell lines KMH2 and L428. CYB5B may be a potential target for antibody-based therapy of HL and aggressive NHL as although cytoplasmic expression is present in reactive lymphocytes, it is not expressed on the cell surface of non-neoplastic lymphocytes or bone marrow precursor cells.

## Background

Human malignant lymphomas are neoplasms arising from lymphocytes at various stages of differentiation, and are currently placed into 2 distinct clinical groupings, namely Hodgkin Lymphoma (HL) and non-Hodgkin Lymphoma (NHL), although, as discussed below, the major groups overlap considerably in terms of cellular origins. NHL is a heterogeneous group of malignant lymphomas comprising over 60 different clinical subtypes, the most common being diffuse large B cell lymphoma (DLBCL), which is an aggressive form, followed by follicular lymphoma (FL) which is usually indolent. T cell lymphomas are generally aggressive but relatively infrequent [1].

HL is subdivided into nodular lymphocyte predominance, and classical types which include 4 subtypes: lymphocyte-rich classical, nodular sclerosis, mixed cellularity and lymphocyte depletion forms [1].

Untreated, all lymphomas are lethal but their natural history varies with each clinical type, stage and other variables both in the neoplasm and the host.

HL had a worldwide incidence of 62,000 cases in 2002; current global estimates are not readily available. Compared with North America and Europe, HL is relatively rare in Japan (age-adjusted incidence of 0.3 per 100,000 males) and China (age-adjusted incidence of 0.2 per 100,000 males). In developing countries, the incidence of the mixed-cellularity (MCHD) and lymphocyte-depleted (LDHD) subtypes of HL is higher than in developed countries. In contrast, the nodular-sclerosis (NSHD) subtype is the most frequent form of HL in developed countries (GLOBOCAN 2002 database. http://www-dep.iarc.fr/).

The common forms of both NHL and HL are derived from clonal B cells at various stages of differentiation and from specific B cell compartments. Whereas B cell derived NHL cases retain many of the B cell lineage specific gene expression programs, the common (classical) forms of HL exhibit loss of expression of B cell lineage genes due to a variety of mechanisms [2-7]. Peripheral T cell lymphomas (PTCL) are derived from post-thymic T cells [8].

B cell-derived Hodgkin and Reed-Sternberg (H/RS) cells of HL and the T cell-derived neoplastic cells of Anaplastic Large Cell Lymphoma (ALCL) constitutively express CD30, a 120 kDa surface phosphorylated glycoprotein [9-11], currently named tumour necrosis factor receptor superfamily, member 8 (TNFRSF8; HUGO Gene Nomenclature Committee). CD30 does not have disease-specificity, as it is an activation-associated antigen expressed by activated T and B cells, HTLV-I or HTLV-II transformed T cells, and EBV-transformed B cells [12,13]. Since anti-CD30 antibodies are not tumour-specific and may target reactive T and B cell subsets [14,15], the creation of antibodies against HL-specific cell surface targets that are not activation-associated markers remains a desirable goal.

Although most patients with HL are cured with first-line therapy, 15%-20% of patients with stage I-II HL and 35%-40% of patients with stage III-IV HL and adverse risk factors relapse after first-line therapy [16,17]. Patients with relapsed or refractory HL are often given second line, salvage chemotherapy followed by high-dose chemotherapy and autologous stem cell transplant (HDCT/ASCT). Patients who fail HDCT/ASCT have a poor outcome [18-21].

Immunotherapy has also been tried in HL patients. As most classical forms of HL express CD30 on the plasma membrane of Reed-Sternberg (RS) cells, the neoplastic cells in HL, various preparations of anti-CD30 antibodies have been tried. These, when used as single-agent therapy in patients who have failed salvage therapy, show only modest effects [22]. Anti-CD30 immunotherapy of HL using radioimmunoconjugates has been associated with severe hematotoxicity [23].

We have recently developed and characterized 2 novel murine monoclonal antibodies, anti-R23.1 and anti-R24.1, that recognize a 21 kDa cell surface molecule expressed by H/RS and ALCL cells, but not by phytohemagglutinin (PHA) activated CD30+ T lymphocytes [24]. The anti-R24.1 antibody also recognises a formalin-resistant epitope expressed in clinical cases of classical HL and aggressive NHL [24]. We have now identified the 21 kDa protein as CYB5B which is expressed on the plasma membrane of lymphoma cells but not normal lymphocytes, reactive lymphocytes or bone marrow precursor cells.

## Results

### Immunoprecipitation and Western Blots After Subcellular Fractionation

**Figure 1:**
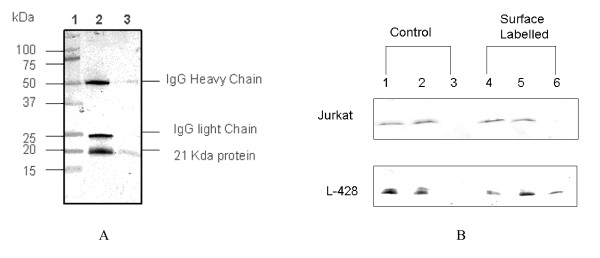
**Immunoprecipitation of the 21 kDa protein**. Panel A: Western-blot analysis of immunoprecipitated 21 kDa protein (probed with anti-R23.1 MAb). Lane 1: MW = Bio-Rad Kaleidoscope molecular weight markers. Lane 2: Protein immunoprecipitated by R23.1. Lane 3: protein immunoprecipitated by R24.1. Both anti-R23.1 and anti-R24.1 MAbs precipitated a protein of ~21 kDa. Panel B: Immunoblots (probed with anti-R23.1 MAb) of subcellular fractions of cell lysates after separation of biotin-labelled surface proteins on NeutrAvidin agarose beads. Lanes 1-3: Unlabelled controls (no biotin label added to surface proteins). Lanes 4-6: Surface biotin labelled protein samples. Lane 1 and 4: Total cell lysate not further fractionated on NeutrAvidin agarose beads. Lanes 2 and 5: Proteins (cytosolic) not bound to the NeutrAvidin agarose beads. Lane 6: Cell surface biotin-labelled proteins eluted from the avidin-beads. The results show that Jurkat and L428 cells contain the protein recognised by anti-R23.1 MAb in the cytosolic fraction (Lane 5) and but only L428 cells express the protein on the cell surface (Lane 6). No protein was detected in eluted fractions from avidin beads in the absence of surface biotin labelling (Lane 3), indicating no evidence of non-specific binding of unlabelled proteins to avidin beads.

Murine monoclonal antibodies anti-R23.1 and anti-R24.1 both precipitated a protein of 21 kDa detected on Western-blots (Figure 1A) as previously described [24]. When L428 HL cells were surface labelled with biotin and then fractionated by binding of biotin-labelled cell surface membrane proteins from cell lysates to NeutrAvidin Agarose, the surface membrane fractions after Western blotting revealed the identical 21 kDa protein. The control cell line, Jurkat, which by flow cytometric analysis was shown to be surface negative (see below), did not demonstrate the presence of the 21 kDa protein in the biotin-labelled cell surface membrane fraction. Both L428 and Jurkat, however, contained the 21 kDa protein in the cytosolic (biotin negative) fraction (Figure 1B).

### 2D-Gel Analysis

**Figure 2:**
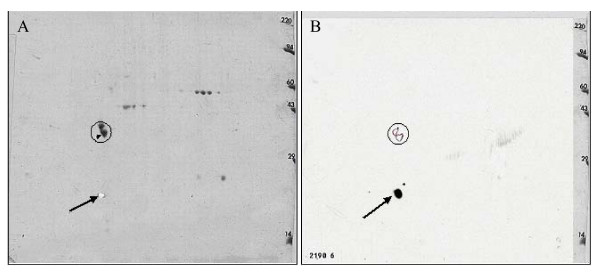
**2D-Gel and Western-blot analysis**. Panel A: Coomassie Blue staining of 2D gel. Panel B: Western-blot analysis with R23.1. The doublet in the circle is tropomyosin used as internal control. The arrow indicates the spot that reacted with R23.1 on the PVDF membrane.

**Figure 3:**
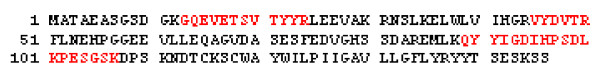
**Mass-Spectrometry data of protein isolated by 2D-gel electrophoresis**. The protein was cleaved by Trypsin from C-term side of KR unless next residue is P. Matched peptides are shown in Bold Red and sequence coverage is 25%.

Two dimensional (2-D) gel electrophoresis followed by blotting onto a PVDF membrane, and probing with antibodies R23.1 and R24.1, and goat-anti-mouse IgG (Horseradish Peroxidase conjugated) revealed only one specific spot on the membrane (Figure 2). Mass-Spectrometric (LC/MS/MS) analysis of the corresponding spot cut out from a duplicate gel detected five peptides which cover 25% of the CYB5B sequence (Figure 3).

### RT-PCR and Sequence Analysis

**Figure 4:**
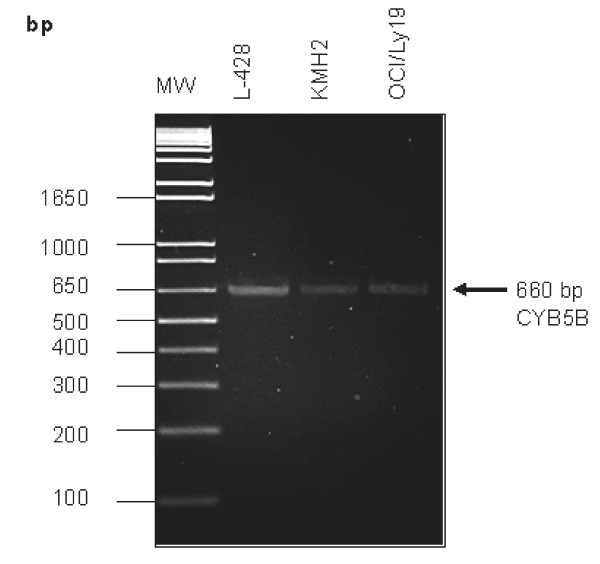
**RT-PCR amplification of *CYB5B***. The *CYB5B *gene was amplified using mRNAs from L428, KMH2 and OCI/LY19 cells as templates and *CYB5B*-F1 and R1 primer pairs. A DNA fragment of 660 bp was detected.

**Figure 5:**
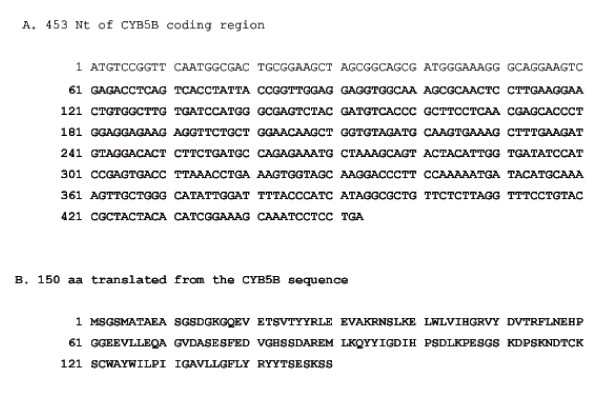
**Sequence analysis of *CYB5B *cDNA**. Panel A: The coding region of *CYB5B *contains no mutation/deletion/insertion. Panel B: 150 AA translated from the *CYB5B *coding region.

RT-PCR amplified a fragment of 660 bp from mRNA sample isolated from KMH2 (HL), L-428 (HL) and OCI/LY19 (diffuse large B cell lymphoma) (Figure 4). Sequence analysis revealed that there is no mutation/deletion/insertion in the coding region of *CYB5B *(Figure 5).

### Protein Array Analysis of R23.1 Antibody Binding Targets

**Figure 6:**
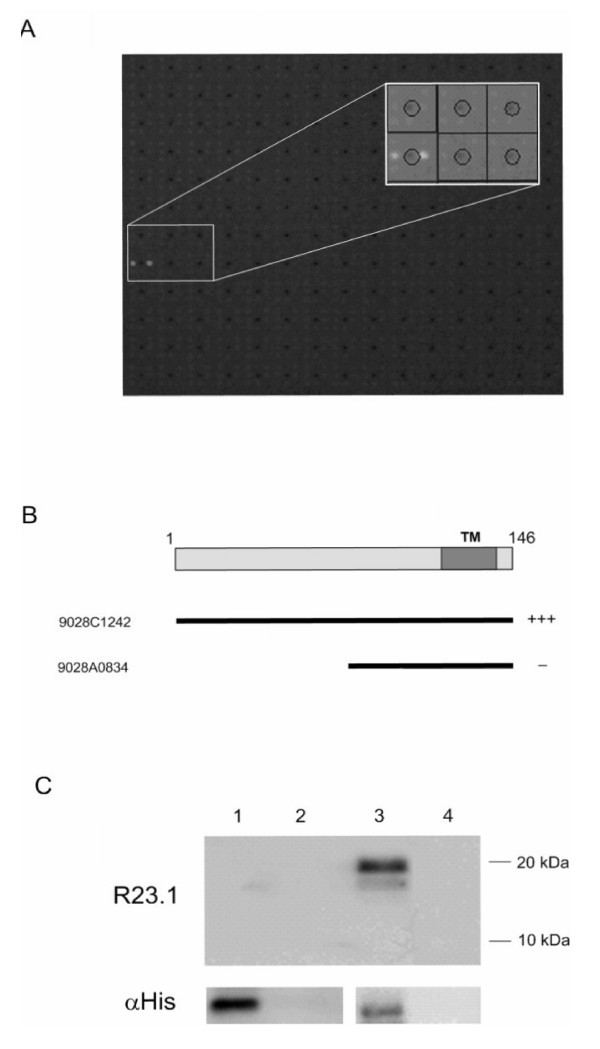
**Protein array identification of the molecular target of R23.1**. Panel A: A PEX protein array containing approximately 16,000 bacterial clones expressing human recombinant His-tagged proteins was screened with the antibody R23.1. The antibody recognised a single clone, 9028C1242. Panel B and C: The clone 9028C1242 (full length CYB5B) and the clone 9028A0834 (expressing the COOH terminal region of CYB5B) were expressed and Western immunoblotted with both R23.1 and an anti-His antibody. R23.1 clearly recognises the approximately 20 kDa full length CYB5B, but not the COOH-region of the protein, revealing the epitope of the antibody to lie within the first 70aa.

A PEX protein array (Imagenes, GmbH) containing approximately 16,000 bacterial clones expressing 10,000 human recombinant His-tagged proteins derived from cDNA libraries from various human tissues, including testis, lymphocytes, fetal brain, lung and colon was screened with the antibody R23.1. The antibody recognised a single clone, RZPDp9028C1242 (Figure 6A), which was derived from a PHA-induced T-lymphocyte cDNA library. Sequencing of this clone revealed it expresses full length CYB5B. The recombinant proteins encoded by clone RZPDp9028C1242 (full length CYB5B) and the clone RZPDp9028A0834 (expressing the COOH terminal region of CYB5B) (Figure 6B) were expressed and immunoblotted with both R23.1 and an anti-His antibody (Figure 6C). R23.1 clearly recognises the approx 21 kDa full-length CYB5B, but not the COOH-region of the protein, revealing the epitope of the antibody to lie within the first 70aa.

### Gene Expression of *CYB5B *in HL, DLBCL and ALCL Cell Lines

**Figure 7:**
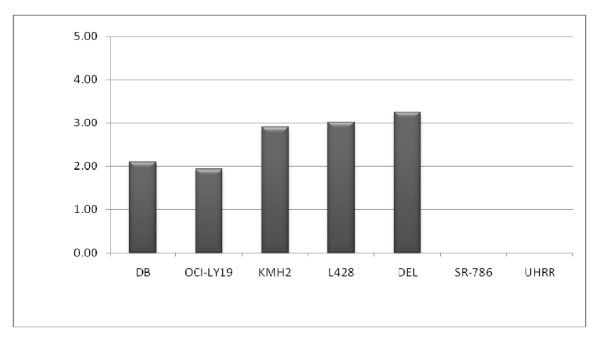
**Quantitative PCR of *CYB5B *expression in lymphoma cells lines**. DLBCL (DB; OCI-LY19), HL (KMH2; L428), and ALCL (DEL; SR-786) cell lines were compared against universal human reference RNA as the calibrator. Relative expression plotted.

Gene expression was first assessed by quantitative PCR. Quantitative PCR showed that HL cell lines KMH2 and L428, as well as ALCL cell line DEL had high relative expression levels using a universal human RNA reference (UHRR) as the calibrator, whereas DLBCL lines DB and OCI-Ly19 had a more modest increase in expression. ALCL cell line SR-786 showed no increase in *CYB5B *mRNA in comparison to UHRR (Figure 7).

**Figure 8:**
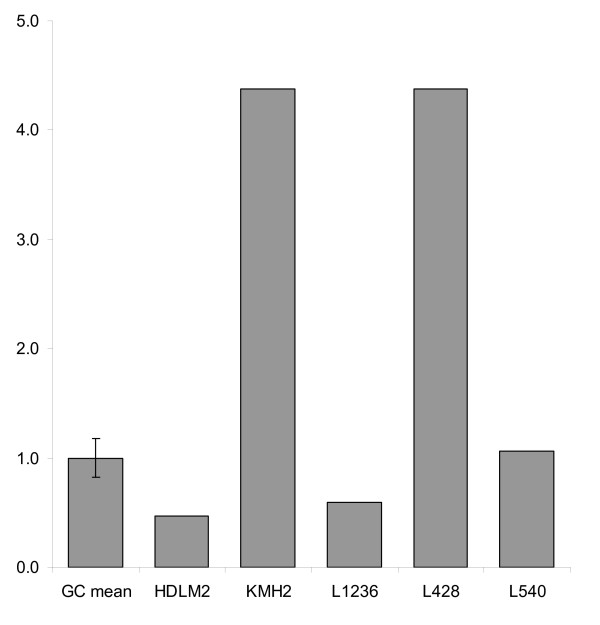
**Relative mRNA expression of *CYB5B *in 5 Hodgkin lymphoma cell lines compared to germinal centre B cells**. Fold difference is shown using normal germinal centre B cells (GC mean) as the control. Two HL cell lines L428 and KMH2 showed over 4-fold increase in *CYB5B *mRNA expression, while 3 HL cell lines HDLM2, L1236 and L540 decreased or similar levels of *CYB5B *mRNA expression (Affymetrix probe set ID 238554_at).

Gene expression of *CYB5B *was also measured in 5 HL cell lines, HDLM2, KMH2, L1236, L428, and L540, using Affymetrix GeneChip^® ^Human Genome U133 Plus 2.0 Arrays. We used microdissected benign germinal centre B cells as the calibrator. Two HL cell lines (KMH2 and L428) had higher expression levels than benign germinal centre B cells, whereas the others (HDLM2, L1236, and L540) had similar or lower expression of *CYB5B *mRNA compared to germinal centre B cells (Figure 8).

### Submegabase Resolution Tiling Array CGH to Assess the *CYB5B *Locus in HL and ALCL Cell Lines

**Figure 9:**
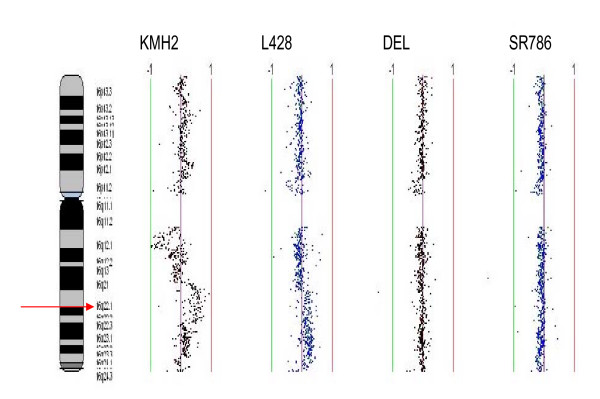
**SMRT-Array CGH of HL and ALCL cell lines**. High resolution karyogram of chromosome 16 showing amplification at 16q22.1 (arrow) in HL cell lines KMH2 and L428 but not ALCL cell lines DEL and SR786.

Submegabase resolution tiling array CGH was used to determine the status of the *CYB5B *locus at 16q22.1 in HL and ALCL cell lines [25]. This showed gains at the *CYB5B *locus at 16q22.1 in HL cell lines KMH2 and L428 (Figure 9), but not in ALCL cell lines DEL and SR786.

### Expression of CYB5 Protein in HL Tissue Microarrays

**Figure 10:**
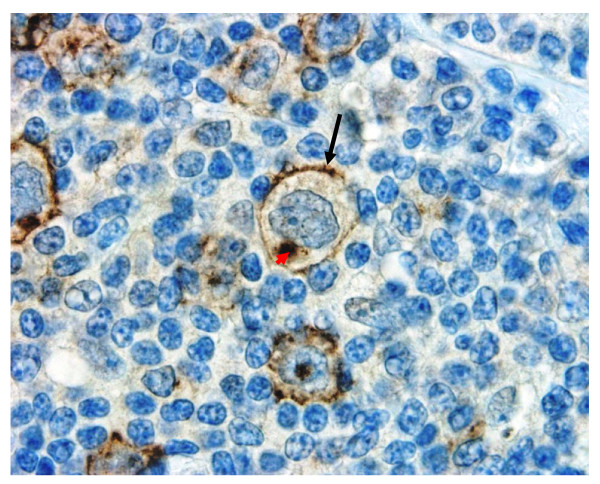
**Immunohistochemical detection of CYB5B protein in HL clinical biopsies**. An example of 2+ membrane positivity for CYB5B protein in Reed-Sternberg cells and mononuclear variants. Note prominent Golgi staining (Red arrowhead) and cell membrane detail showing a ruffled pattern (black arrow). Magnification × 600.

**Figure 11:**
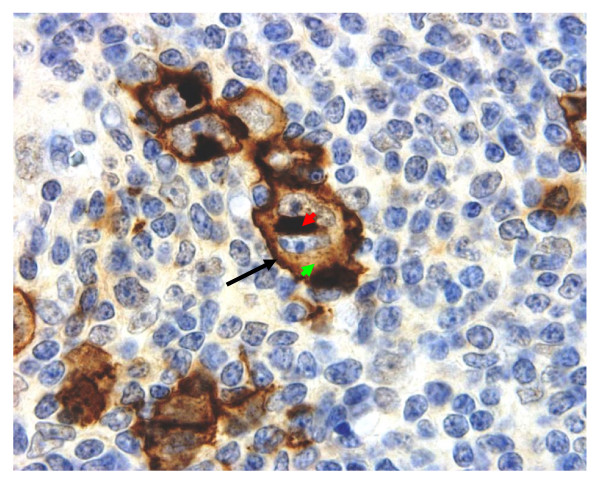
**Immunohistochemical detection of CYB5B protein in HL clinical biopsies**. An example of intense (3+) positivity for CYB5B on the cell membrane (black arrow) and Golgi (red arrowhead) regions of Reed-Sternberg cells and mononuclear variants. Note speckled cytoplasmic staining pattern (green arrowhead). Magnification × 600.

A tissue microarray (TMA) of 199 cases of Hodgkin Lymphoma was assembled in duplicate. Of these, 9 cases were of nodular lymphocyte predominance (NLPHL) type, and the rest (190) of classical types. Reed-Sternberg cells and their variants were present in the TMA sections for evaluation in 180 cases. As summarised in Table 1, 37% of cases of classical HL showed moderate (2+) to intense (3+) membrane positivity. When cases with both cytoplasmic staining and any intensity for membrane staining were included, 87% of all Classical types of HL were positive for CYB5B expression. Examples of 2+ and 3+ membrane staining patterns are shown in Figures 10 and 11, respectively. In both examples, cytoplasmic staining is limited to the Golgi zone and a cytoplasmic speckled pattern that might reflect mitochondrial staining (this will require verification by confocal microscopy). None of the cases of NLPHL showed membrane positivity but 44% (4 of 9 cases) showed weak diffuse cytoplasmic staining.

**Table 1:** CYB5B Expression in Hodgkin Lymphoma Tissue Microarray Sections

	Membrane Positivity 2+ to 3+	Cytoplasmic and all membrane positivity
Evaluable cases	180	180

NLPHL	9	9

Classical HL	171	171

Positive Cases Of Classical HL	64 (37%)	148 (87%)

Positive Cases Of NLPHL	0 (0%)	4 (44%)*

*Cytoplasmic only.

### Expression of CYB5B Protein in Reactive Lymph Nodes

**Figure 12:**
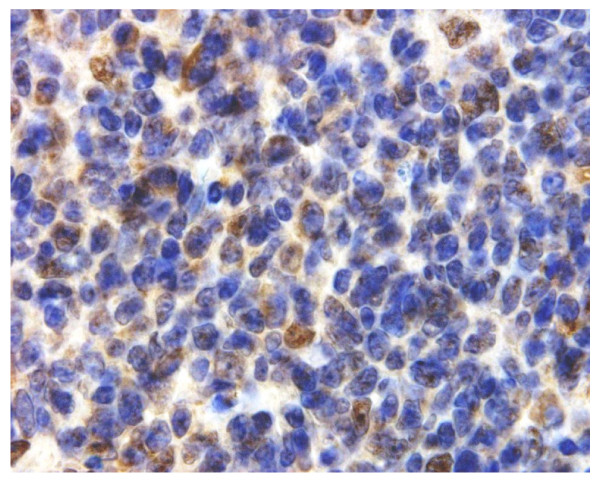
**Immunohistochemical detection of CYB5B protein in reactive lymph node**. Germinal centre centrocytes with cytoplasmic expression of CYB5B protein. Membrane staining is not evident. Magnification × 400.

**Figure 13:**
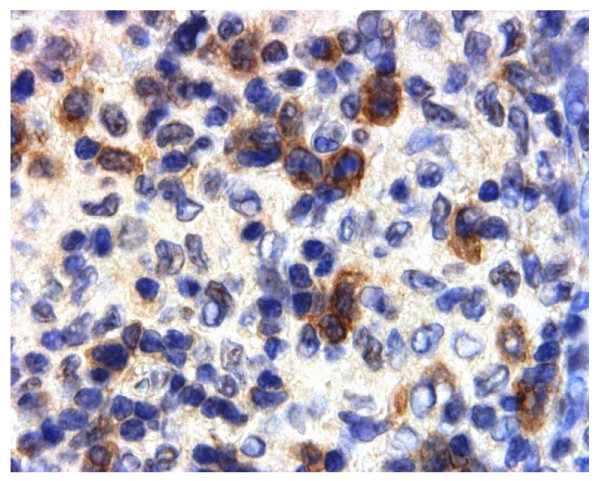
**Immunohistochemical detection of CYB5B protein in reactive lymph node showing dermatopathic change**. Intense cytoplasmic staining for CYB5B in T-zone lymphocytes. Membrane staining is not evident. Magnification × 600.

Immunohistochemistry for CYB5B protein was conducted on 6 reactive lymph nodes. These showed no evidence of membrane positivity. However, variable diffuse cytoplasmic staining was noted in centrocytes (Figure 12) and in isolated cells in the T zone. In one case of dermatopathic lymphadenopathy, a condition which is usually due to a reactive T cell response to various types of dermatoses [26], intense cytoplasmic staining was noted in the activated T cell zone in isolated cells (Figure 13). No speckled pattern of staining in the cytoplasm was noted unlike in HL cases.

### Flow Cytometric Analysis of Surface and Cytoplasmic CYB5B Protein Expression in Jurkat Cells

**Figure 14:**
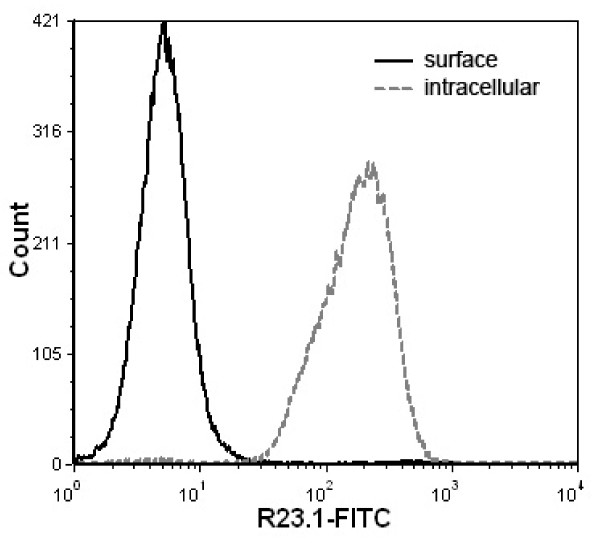
**Flow cytometric detection of CYB5B in Jurkat cells**. Histograms show the fluorescence intensity distribution of R23.1-FITC-stained Jurkat cells, stained either for cell-surface R23.1 on viable cells (black solid line) or intracellular R23.1 within fixed cells (gray dashed line). There is no detectable surface labelling whereas intracellular labelling is evident.

**Figure 15:**
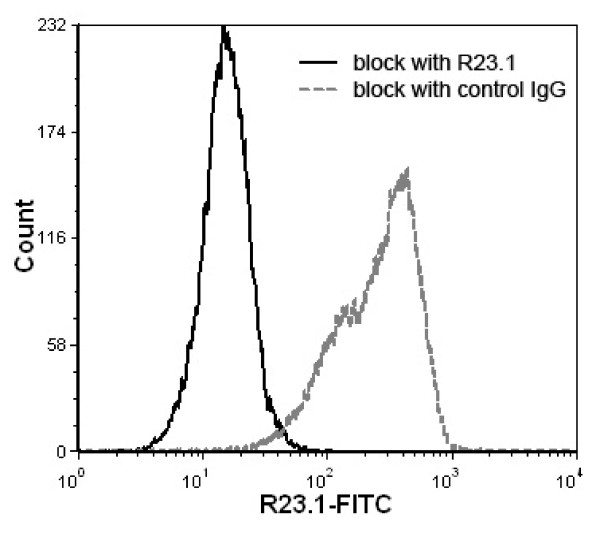
**Flow cytometric detection of CYB5B in Jurkat cells**. Histograms show the fluorescence intensity distribution of R23.1-FITC-stained, fixed/permeabilized Jurkat cells blocked either with control mouse IgG (black solid line) or unlabelled R23.1 (gray dashed line). Whereas blocking with unlabelled R23.1 mAb inhibited intracellular binding of R23.1-FITC, a similar effect was not seen with control IgG.

Jurkat cells were previously reported to have no surface expression of CYB5B protein by flow cytometry [24]. This was also confirmed by subcellular fractionation of Jurkat cell lysates (Figure 1A). We also used permeabilized Jurkat cells to demonstrate the presence of intracellular CYB5B protein in the absence of plasma membrane CYB5B (Figure 14) by flow cytometry. Cytoplasmic labelling of CYB5B by mAb R23.1-FITC was blocked by unlabelled mAb R23.1 but not mouse isotype control IgG_1 _(Figure 15) indicating that this was not a result of non-specific binding of anti-R23.1 to permeabilized cells.

### Bone Marrow CD34+ Precursor Cells Do Not Express CYB5B at the Plasma Membrane

**Figure 16:**
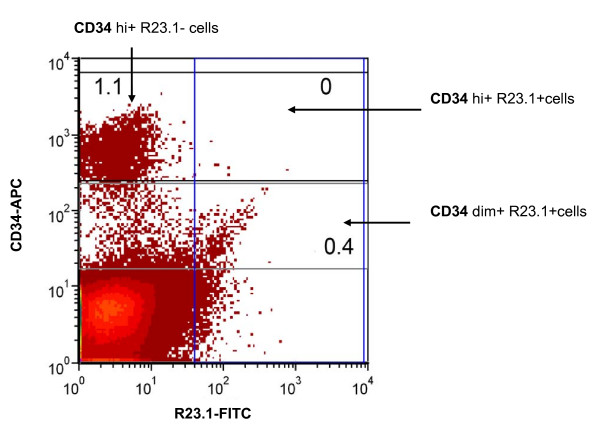
**Flow cytometric detection of CYB5B in bone marrow precursor cells**. Bone marrow cells were gated for viable lymphocytes using forward vs. side scatter and propidium iodide exclusion, and then analysed for surface CD34-APC and R23.1-FITC expression. Numbers indicate the percentages of the total cells in the plot falling into the indicated gates. In this example, 0.4% of cells expressed dim CD34 and R23.1 and none coexpressed CD34 hi (bright) and R23.1.

**Figure 17:**
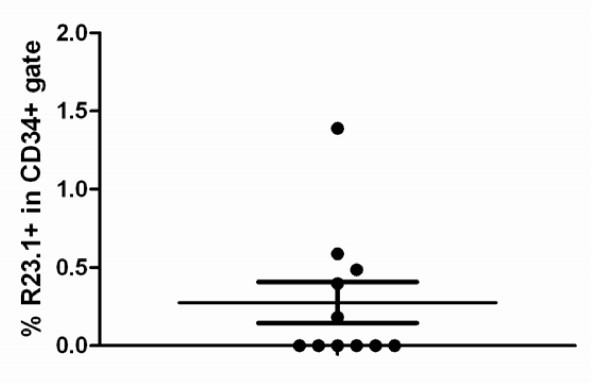
**Results of flow cytometric detection of CYB5B in bone marrow precursor cells**. Vertical scatter plot of the incidence of R23.1+ cells in the CD34-hi gate. Data shown are the percentage of all CD34-hi cells which are also R23.1+ from 9 different normal bone marrow samples. Mean percentage ± s.d. = 0.3 ± 0.4.

Dual colour flow cytometry was used to assess the plasma membrane expression of CYB5B on viable CD34+ cells in normal bone marrow samples (Figure 16). This showed that there is negligible expression of CYB5B by CD34+ cells, with most samples showing 0% positivity (mean% ± s.d. = 0.3 ± 0.4; Figure 17).

### RT-PCR of *CYB5B *in T and B Cell Enriched Fractions from Reactive Lymph Nodes, Tonsils, Normal Peripheral Blood Lymphocytes and Bone Marrow Samples

**Figure 18:**
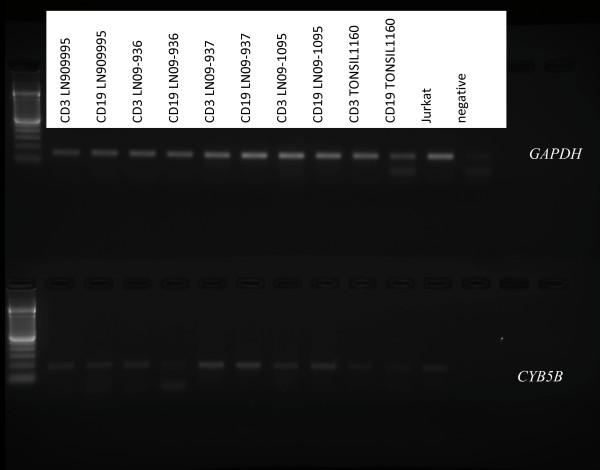
**Relative levels of *CYB5B *mRNA expression in T and B lymphocytes isolated from reactive lymph nodes and tonsils**. RT-PCR for *GAPDH *and *CYB5B *from RNA extracted from lymph node (LN) and tonsil lymphocytes enriched for CD3+ T cells and CD19+ B cells showed no increase in *CYB5B *mRNA in comparison to *GAPDH*.

**Figure 19:**
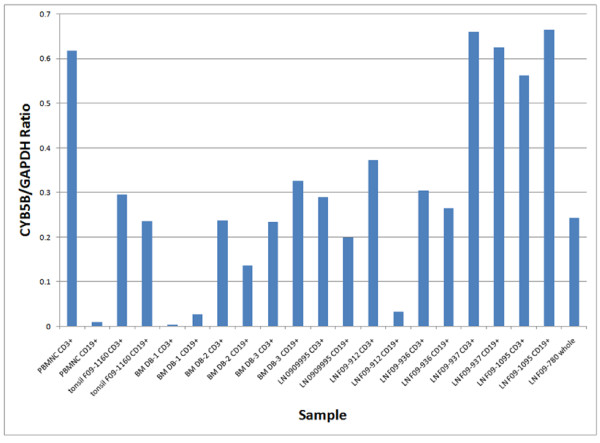
**Quantification of *CYB5B *RT-PCR data from clinical samples**. cDNA was prepared from both CD3-selected and CD19-selected cells from normal peripheral blood mononuclear cells (PBMNC), reactive tonsils, normal bone marrow (BM) and reactive lymph node samples. PCR was performed using primers to the *GAPDH *housekeeping gene. Bands from the *GAPDH *PCR were used to normalize loading for the CD3+ and CD19+ cDNA pools for each specimen. Then, PCR was performed in duplicate for *CYB5B *and *GAPDH*, using the same amount of cDNA and the same number of cycles. Bands were quantified using spot densitometry and the ratio of *CYB5B/GAPDH *signal was calculated. Values shown are from a typical experiment; each sample was analyzed by at least two independent PCRs. In none of the samples did the *CYB5B *signal exceed that of *GAPDH*.

*CYB5B *expression was detected by RT-PCR in the CD3+ enriched fractions and in the CD19+ enriched fractions from reactive lymph nodes, tonsils, peripheral blood mononuclear cells, and normal bone marrow samples, but at lower levels than housekeeping gene *GAPDH *(Figures 18 and 19), indicating no differential increase of *CYB5B *mRNA in benign lymphocytes even in reactive conditions.

## Discussion

We recently described a novel 21 kDa cell surface and cytoplasmic protein expressed by HL and ALCL cell lines, other CD30+ non-Hodgkin lymphoma cell lines, HRS cells in HL clinical biopsies, and the neoplastic cells in biopsies from ALCL and aggressive NHL patients[24]. Using two independent methods, we now show that the protein is identical to a poorly characterised molecule which has been postulated to be outer mitochondrial membrane protein Cytochrome B5B (CYB5B) based on incomplete (45.8%) homology to Cytochrome B5A, an endoplasmic reticulum protein [27]. The first method was a classic approach, first separating all proteins from HL cell line L428 lysates by 2-dimensional gel electrophoresis, detecting the 21 kDa protein of interest by immunoblotting with anti-R23.1 mAb and then cutting out the identical spot from a duplicate gel and subjecting the extracted protein to LC/MS/MS for peptide identification after trypsin digestion. The resulting peptide sequence matched that of the predicted CYB5B protein sequence. The second method involved screening for anti-R23.1 mAb binding to a protein array containing approximately 16,000 bacterial clones expressing 10,000 human recombinant His-tagged proteins derived from cDNA libraries from various human tissues, including testis, lymphocytes, fetal brain, lung and colon. The antibody recognised a single clone, RZPDp9028C1242, which was derived from a PHA-induced T-lymphocyte cDNA library. There was no cross reactivity with any other clone on the array, indicating high specificity for the protein expressed by clone RZPDp9028C1242. Sequencing of this clone revealed that it expresses full-length CYB5B.

Since we had previously reported that PHA-activated T cells do not express the 21 kDa protein at the cell surface [24], the detection of CYB5B in a PHA-induced T-lymphocyte cDNA library derived protein array may seem like a contradiction. However, it should be pointed out that PHA activated T cells contain intracellular CYB5B, but do not express it on the cell surface, since propidium iodide non-excluding (i.e. non viable and therefore naturally permeabilized due to loss of membrane integrity) PHA activated T cells bind anti-R23.1 mAb (unpublished observations). This is also seen in T cell derived Jurkat cells which contain intracytoplasmic CYB5B but lack plasma membrane expression of this protein, as we have shown in this paper.

We next looked at the relative expression of *CYB5B *mRNA in cell lines. Quantitative PCR, using universal human reference RNA (UHRR; Stratagene) as the calibrator, showed *CYB5B *gene expression was increased in HL and NHL cell lines with the highest expression in ALCL line DEL, followed by HL lines L428 and KMH2. Surprisingly, ALCL cell line SR-786 showed no increase in CYB5B expression. This was puzzling at first as we have previously reported that SR-786 cells are positive for the membrane protein labelled by mAb anti-R23.1 [24]. UHRR was developed at Stanford University by the Botstein/Brown laboratory and is commercially available from Stratagene. The original set of cell lines used to create the pooled reference human RNA has not been fully documented in indexed publications [28] but includes 3 different malignant lymphoid or histiocytic cell lines (histiocytic lymphoma, presumed of macrophage origin, lymphoblastic lymphoma and a plasmacytoma which is of B cell origin) [29]. It is possible therefore that UHRR contains a relatively high concentration of *CYB5B *mRNA and, if used as the calibrator, may lead to an underestimation of *CYB5B *in cell lines such as SR-786 if it has similar levels of the *CYB5B *mRNA.

We therefore used an additional platform to assess *CYB5B *gene expression levels in HL cell lines using the Affymetrix GeneChip^® ^Human Genome U133 Plus 2.0 Arrays and used RNA from pooled benign germinal centre B cells isolated from reactive lymph nodes by microdissection as the calibrator. Since the HL cell lines are derived from B cells, we felt benign reactive germinal centre B cell-derived RNA would be suitable as a calibrator. We noted, using this assay, that *CYB5B *expression is increased in HL cell lines KMH2 and L428, but not HDLM2, L1236, and L540. Again, there remains a possibility that reactive germinal centre B cells have increased expression of *CYB5B*, and this might lead to underestimation of relative mRNA levels in the cell lines tested. We then looked at *CYB5B *mRNA in fresh samples of normal T and B cells harvested from peripheral blood, reactive lymph nodes, tonsils, and normal bone marrow samples using *GAPDH*, an ubiquitous housekeeping gene, as the calibrator. There was no evidence of overexpression of *CYB5B *mRNA in any of these samples. Thus the data from the Affymetrix experiments may be a true reflection of increased *CYB5B *mRNA in 2 of 5 HL cell lines with no bias created by the calibrator.

Since there was evidence that HL cell lines KMH2 and L428 consistently showed increased expression of *CYB5B *mRNA regardless of the assay method used, and ALCL cell line DEL also showed an increase at even higher levels than the 2 HL cell lines, one of the possible explanations was amplification of the *CYB5B *gene in these cell lines. Array CGH using a submegabase resolution tiling array revealed gains in the *CYB5B *locus in HL cell lines KMH2 and L428, but not in ALCL cell line DEL. Thus, although gene amplification may be a mechanism of increased *CYB5B *mRNA levels in KMH2 and L428 cells, this is unlikely in DEL cells. The status of amplification of the gene in clinical samples will be necessary to determine if this is a clinically relevant observation.

There is very little published literature on human CYB5B. Prior publications regarding the two membrane-anchored isoforms of mammalian microsomal and outer mitochondrial membrane Cytochrome *b5 *used the older nomenclature of MC *b5 *and OM *b5 *[20] or cyt-*b5 *types 1 and 2 [27], respectively. The Cytochrome B5 isoforms in mammals are electron transfer molecules which have a heme binding extramembrane domain (~100 residues) and a membrane anchoring domain contained within the 10 polar C-terminal residues [21-23]. Much of the prior data on CYB5B relates to the rat outer membrane protein rOM *b5 *which was isolated from rat liver as a 92 residue fragment representing the heme-binding domain [30] and the cloned gene showed it has 146 residues [31]. Aliases include OMB5; CYB5-M; CYPB5 M; CYPB5 M; CYTB5, TYPE 2 and DKFZp686 M0619 [OMIM: *611964; GenBank:AK291576; NM_030579; Gene ID 80777]. The *CYB5B *gene is located on chromosome 16 (16q22.1; [OMIM: *611964]). By similarity alone, but without experimental evidence, human CYB5B has been so named due to partial (45.8%) homology to CYB5A [27], and high homology to rat outer membrane of mitochondrial protein rOM *b5 *(74% sequence identity) [Ensembl: ENSRNOG00000011142].

B5-domain proteins can be divided into two main classes - those with a heme-binding cytochrome *b5 *fold including Cytochrome *b5*, bacterial cytochrome *b558*, flavocytochrome *b2*, sulfite oxidase, nitric reductase and other oxidoreductases, and non-heme-bindings proteins such as chitin synthases, probable E3 ubiquitin-protein ligase HERC2 and the membrane-associated progesterone receptor family MAPR [32]. MAPR family proteins include neuron derived neurotrophic factor NENF or neudesin [33], progesterone receptor membrane component 1 (PGRMC1) [32] and progesterone receptor membrane component 2 (PGRMC2) [34]. Possible paralogs include the aquaporin family of water transport proteins (Ishibashi,K.; Cytochrome b5 and aquaporins share the last transmembrane amino acids sequence; unpublished; http://www.uniprot.org/uniprot/O43169), and HERC2 ubiquitin transferase proteins and a number of chitin synthases as mentioned above [35]. The KEGG site also lists 10 paralogs including cytochrome b5 type A (microsomal), fatty acid desaturase 2, fatty acid desaturase 1, sulfite oxidase, cytochrome b5 reductase 4, fatty acid 2-hydroxylase, fatty acid desaturase 3, hect domain and RLD 2, cytochrome b5 domain containing 1, and aquaporin 4 http://ssdb.genome.jp/ssdb-bin/ssdb_paralog?org_gene=hsa:80777.

As most of the related molecules are secreted proteins (NENF) or endoplasmic reticulum proteins (PGRMC1 and 2), the presence of CYB5B at the plasma membrane of HRS and ALCL cells is unexpected. However, endoplasmic reticulum tail-anchored proteins with short hydrophobic domains such as the CYB5A isoform, if lengthened by any inserted sequence of as few as 5 amino acids, can be induced to relocate to the plasma membrane [36,37]. When we checked the *CYB5B *gene in HL cells for mutations that might lengthen the hydrophobic domain we found none, thus this explanation does not appear to hold true in human lymphoma cells. The presence of unmutated CYB5B in the plasma membrane of HL and NHL cells suggests that the protein is likely to be translocated to the plasma membrane by an alternate mechanism. Such mechanisms could include other post-translational changes that result in altered hydrophobicity [38,39], differences in glycosylation [38] or interaction with one or more molecular chaperones or heat shock proteins [40,41].

Although the function of human CYB5B protein is poorly characterised at this time and the mechanism of its trafficking to the plasma membrane of Hodgkin and aggressive non-Hodgkin lymphoma cells is unexplained, the protein could be a useful target for antibody-based therapy as it is not expressed at the plasma membrane of normal lymphocytes, reactive lymphocytes, or bone marrow precursor cells. Toxicity, therefore, might be minimal if anti-CYB5B antibodies are used for targeted therapy of CYB5B surface positive lymphomas.

## Conclusions

Anti-R23.1 and anti-R24.1 MAbs recognise a 21 kDa plasma membrane and cytoplasmic protein expressed by HL and aggressive NHL that is identical to CYB5B, a protein that has been putatively named as an outer membrane mitochondrial protein based on partial similarities to CYB5A, an endoplasmic reticulum tail-anchored protein and high sequence identity with rat outer membrane mitochondrial protein rOM b5. The encoding gene is unmutated in HL cell lines. The presence of unmutated CYB5B in the plasma membrane of HL and NHL cells suggests that the protein is likely to be translocated to the plasma membrane by a hitherto unknown mechanism.

*CYB5B *gene expression is increased in HL and NHL cell lines with the highest expression in ALCL line DEL, followed by HL lines L428 and KMH2. This is associated with *CYB5B *amplification in HL cell lines KMH2 and L428, but not in ALCL cell line DEL. *CYB5B *mRNA expression is not increased in reactive lymphocytes but the CYB5B protein is present in detectable quantities in the cytoplasm of such cells. The protein is not expressed at the plasma membrane of bone marrow precursor cells, but using immunohistochemistry on tissue microarrays of clinical cases of HL, plasma membrane and/or cytoplasmic expression was detected in HL neoplastic HRS cells in 87% of clinical cases, with intense membrane staining in 37% of clinical cases. The plasma membrane expression of CYB5B protein in neoplastic cells in HL is potentially useful for antibody-based targeted therapy in a subset of patients especially those with high levels of membrane expression. Since normal lymphocytes and bone marrow precursor cells lack surface expression of CYB5B, little or no toxicity should be observed during anti-CYB5B immunotherapy.

## Methods

All experimental protocols were reviewed and approved by the University of British Columbia (UBC) Research Ethics Board (REB) (Certificate #H06-60016).

### Cell Lines, Cell Culture Conditions

The human HL-derived cell lines KM-H2, L-428 and the ALCL cell lines DEL and SR-786 were obtained from the Deutsche ammlung von Mikrooganism und Zellkulturen GmbH (Braunschweig, Germany). All cell lines were cultured in RPMI 1640 supplemented with 10% heat-inactivated fetal bovine serum (Cansera Inc., Etiobicoke, ON, Canada), L-glutamine and penicillin and streptomycin in a humid environment of 5% CO2 at 37°C to a density of 10^6 ^cells/ml.

### Cell Lysate Preparation

HD lymphoma cell line, L-428, was cultured in IMDM complete media (10% FBS, with penicillin/streptomycin/fungizone, Gibco) for 48 hrs. The cultured cell was harvested and pelleted at ~1000 g for 5 minutes. The cells were washed in PBS (pH7.2) for 3 times and lysed in Mammalian protein extraction buffer supplied by Pierce. The samples were incubated on ice for 10 minutes and pelleted at 10,000 × g for 10 min. The supernatant was transferred to fresh tubes.

### Formation of the Antibodies and Protein G Beads Complex

100 μl of protein G beads (50%) (Pierce) were added to each micro-centrifuge tube. The beads were pelleted at <2000 g for 30 seconds and washed in 1 ml of PBS once. 200 μl of PBS and 5 μg of antibodies were added to each tube. The beads were incubated at room temperature for at least 2 hrs and washed in PBS twice. Normal mouse IgG was coupled to the protein A beads in the same way and these beads are used for pre-clearing the lysates.

### Antigen Capture

400 μl of the sample was incubated with normal mouse IgG-protein-A beads for 1 hr at room temperature and transferred into the tube containing mAb-protein A complexes. The beads were then incubated at 4°C overnight and washed in 1 ml of PBS for 3 times. The beads were resuspended in 1 ml of PBS and 50 μl of beads were transferred into another 1.5 ml tube. The beads were pelleted and as much buffer as possible was removed. The original tube was stored at -20°C.

### SDS-Page and Western-Blot Analysis

25 μl of the 1 × SDS sample buffer was added to the second tube and heated at 100°C for 5 min. The tube was centrifuged 13000 ×g briefly and the sample was loaded to 4-20% Tris-Glycine gel (Bio-Rad). The samples were separated at 120 V for 80 min. The gels were equilibrated in pre-chilled 1× transfer buffer and the proteins were then transblotted onto a nitrocellulose membrane at 90 volts for 60 min. Western-blot analysis with R24.1/R23.1 was performed by blocking the membrane in 5% non-fat-dry milk, and probing with primary antibody (R24.1/R23.1) and secondary antibody (goat-anti-mouse IgG-AP) sequentially. The protein band which reacted with the antibody was visualized in BCIP/NBT substrate. The original tube was submitted to Kendrick Laboratories, Inc. (Madison, WI, USA) for 2D-gel analysis after the Western-blot analysis confirmed the candidate protein was pulled down by the antibody.

### Surface Protein Labelling

Surface protein labelling with biotin was performed with the Pierce Cell Surface Protein Isolation Kit as per the manufacturer's instructions (Thermo Fisher Scientific, Fisher Canada, Nepean, Ontario). Briefly, Jurkat and L-428 cells were cultured in RPMI 1640 (STEMCELL Technologies, Vancouver, British Columbia, Canada) with 10% FBS, and 1× Antibiotic Antimycotic (GIBCO Invitrogen Canada Inc. Burlington, Ontario) for 48 hrs. Cells were harvested and washed twice in ice-cold PBS and counted. 2.5 × 10^7 ^cells were used for surface labelling. One vial of Sulfo-NHS-SS-Biotin was dissolved in 48 ml of ice-cold PBS. 24 ml of the biotin solution were added to each labelling tube. PBS was used to replace the Sulfo-NHS-SS-Biotin for the control tubes. The tubes were placed on a rocking platform for 30 minutes at 4°C. 500 μl of Quenching Solution were added to each tube to quench the reaction. 20 ml of TBS were added to each tube and the cells were centrifuged at 500 × g for 3 minutes. The cells were washed twice in 20 ml of TBS and lyzed in 500 μl of lysis buffer with protease inhibitor. The samples were transferred into 1.5 ml microcentrifuge tubes and incubated on ice for 30 min. The samples were vortexed every 5 min for 5 seconds and centrifuged at 10,000 g for 2 min at 4°C. 500 μl of the NeutrAvidin Agarose slurry were added to the spin column and washed with 400 ul of Wash Buffer for 3 times. The supernatants were incubated in the columns for 60 min at room temperature on with end-over-end mixing using a rotator. The columns were centrifuged at 1,100 × g for 1 min and the flow-throughs were transferred to fresh tubes. The columns were washed in 500 μl Wash buffer 4 times. 400 μl of SDS-Page sample buffer were added to each column and the columns were incubated at room temperature for 1 hr with the end-over-end mixing. The columns were centrifuged at 1,000 × g for 3 minutes and the eluted samples were analyzed on 4-20% Tris-HCl gel (Bio-Rad Laboratories (Canada) Ltd. Mississauga, Ontario). SDS-PAGE and Western-blot analysis was performed following the same protocol as described above.

### 2D-Gel Analysis and Coomassie-Blue Staining

Two-dimensional electrophoresis was performed according to the carrier ampholine methods [42] as follows: Isoelectric focusing was carried out in glass tubes of inner diameter 2.0 mm using 2% pH 3.5-10 (GE Healthcare, Piscataway, NJ) for 9600 volt-hrs. One μg of an IEF internal standard, tropomyosin, was added to each sample. This protein migrates as a doublet with lower polypeptide spot of MW 33,000 and PI 5.2; an arrow on the stained gels marks its position. The enclosed tube gel pH gradient plot for this set of ampholines was determined with a surface pH electrode. After equilibration for 10 min in Buffer 'O' (10% glycerol, 50 mM dithiothreitol, 2.3% SDS and 0.0625 M Tris, pH 6.8) the tube gels were sealed to the top of stacking gels on top of 10% acrylamide slab gels (0.75 mm thick) and SDS slab gel electrophoresis carried out for about 4 hrs at 12.5 mA/gel. The following proteins (Sigma Chemical Co., St. Louis, MO) were added as molecular standards to a well in the agarose that sealed the tube gel to the slab gel: myosine (220,000), phosphorylase A(94,000), catalyse (60, 000), actin (43, 000), carbonic anhydrase (29,000) and lysozyme (14, 000). These standards appear as bands at the basic edge of the Coomassie blue-stained 10% acrylamide slab gel. The gel was dried between sheets of cellophane with the acid end to the left.

After slab gel electrophoresis, the gel for blotting was transferred to transfer buffer (12.5 mM Tris, pH8.8, 86 mM Glycine, 10% MeOH) and transblotted onto PVDF paper overnight at 200 mA at approximately 100 volts/two gels. Gel ID numbers are indicated in the lower acid corner of the membrane.

Western-blot analysis was performed on PVDF paper. The PVDF paper was incubated in 5% non-fat dry milk for 2 hrs followed by an incubation in primary antibody (1:1000 diluted R24.1/R23.1) at 4°C overnight. The PVDF was washed in PBS 4 times and then incubated in goat-anti-mouse IgG-HRP conjugate (1:2000) at room temperature for 2 hrs. The paper was washed 4 times in PBS and incubated in 10 ml of SuperSignal West Pico Chemiluminescent substrate (Pierce Biotech., Rockford, IL) fro 10 min. A Kodak BioMAx MS film was exposed to the PVDF paper in a film cassette. The protein spot on a duplicate dried 2D gel was cut and submitted to Protein Chemistry Core Facility in Columbia University (New York, New York) for LC/MS/MS analysis.

### RT-PCR and Sequence Analysis

cDNAs of *CYB5B *were amplified from mRNA with Clontech's Titanium One Step RT-PCR kit (Clontech, Mountain View, CA, USA). The forward primer is 5'-CTCAAGGAAAGTAGTCGCGG-3', which starts at the first nucleotide of 5' end of the mRNA. The reverse primer is 5'-AACTCTTGGCCTCAAGCGA-3', which starts at the nucleotide 660. A product of 660 bp is expected to be amplified using this primer pair. The amplified RT-PCR products were gel purified with Spinprep Gel DNA kit (Novagen, Gibbstown, NJ, USA). The purified samples were submitted to MacrogenUSA Inc. (Rockville, MD, USA) for sequencing.

### Protein Arrays

The protein arrays used in this study are the PEX arrays from Imagenes (Berlin). Each array contains 16,000 *Escherichia coli *His-tagged expression clones from a variety of expression libraries arrayed in a 3 × 3 pattern on PVDF membranes. The expression vector used is pQE30NST (GenBank Accession No. AF074376) and it is transformed into *E. coli *strain SCS1 (Stratagene) [43,44]. Sequence information for individual clones recognized by antibodies was provided by Imagenes.

### Antibody Screening

Prior to antibody screening, the arrays are prepared by removing the dried colonies from the PVDF membranes with tissue paper and TBSTT (TBS supplemented with 0.05% Tween 20 and 0.5% TritonX-100). This is followed by three washes, for 20 min each, in TBST (TBS supplemented with 0.05% Tween 20) and blocked with 3% (w/v) milk powder in TBST for 3 h at room temperature, the filters were incubated overnight with the monoclonal antibody R23.1 [24] at a 1:1000 dilution in 2% BSA/TBST. Primary antibody incubation was followed by three washes of 20 min each in TBST, and the protein arrays were then incubated with the secondary antibody, alkaline phosphatase-conjugated (AP) anti-mouse IgG (A1418; Sigma,) for 1.5 h. Following three washes for 20 min each in TBST, the filters were incubated for 10 min in AP buffer (1 mM MgCl_2_, 100 mM Tris-Cl, pH 9.5) and 5 min in 0.125 mM Attophos in AP buffer. Filters were illuminated with long-wave UV light (460 nm EPI) and images were taken using a high resolution CCD camera (Fuji LAS 3000; Fuji Film Global)). Image analysis was performed using VisualGrid (VisualGrid™ is available as a free download from (GPC Biotech, Munich, Germany) and the identities of detected clones were confirmed by sequencing.

### Recombinant Protein Expression and Purification

The *E. coli *expression clones 9028C1242 and 9028A0834 from the PEX library were grown in 5 ml liquid cultures and protein expression induced with 1 mM IPTG and purified as previously described using nickel affinity chromatography [43,45]. The size of each purified protein was determined using SDS PAGE and Coomassie staining.

### Western Blotting

The purified proteins were separated by SDS PAGE and transferred onto PVDF membranes at 100 V for 1 1/2 hr. The membrane was blocked for 2 hr in 3% Marvel/TBST, washed in TBS (3 × 10 min) and incubated for 2 hr with the primary antibody as described above. Antibody binding was detected using an alkaline phosphatase-conjugated anti-mouse, or anti-rabbit, IgG secondary antibody (Sigma) and visualized on a Fuji LAS 3000 imager.

### SMRT-Array CGH and Gene Expression Profiling

Tiling path array comparative genomic hybridization was performed as described previously [25]. Genomic DNA was extracted via standard proteinase K/RNAse treatment and phenol-chloroform extraction.

Briefly, 200 ng of test and reference DNA were separately labelled with Cyanine3-dCTP and Cyanine5-dCTP (Perkin Elmer Life Sciences Inc., Boston, MA, USA), respectively, via random priming at 37°C for 16-18 h in the dark. Labelled sample and reference DNA probes were combined, precipitated, re-suspended, blocked and hybridized onto BAC arrays containing 26,819 clones spotted in duplicate on a single slide. After hybridization, arrays were washed, rinsed, and dried using an oil free air stream. Slides were scanned using a charge-coupled device (CCD) based imaging system (arrayWoRx eAuto, Applied Precision, Issaquah, WA, USA). Images were analyzed using SoftWoRx Tracker Spot Analysis software (Applied Precision). The ratios were normalized using a stepwise normalization technique to remove any biases added from non-biological sources.

Custom software (SeeGH) [46] was used to visualize normalized data as log_2 _ratio plots. Genomic alterations were classified as homozygous deletions, loss, normal, gain, and amplification for ratios < -1.0, -1.0 to -0.15, -0.15 to 0.15, 0.15 to 1.0, and >1.0 respectively [47]. SeeGH is now freely available from http://www.flintbox.ca/technology.asp?page=312.

### Relative Expression of *CYB5B *in Cell Lines Using qRT-PCR and Affymetrix GeneChip^® ^Human Genome U133 Plus 2.0 Arrays

Validation of the expression of *CYB5B *was carried out by quantitative real-time PCR (qRT-PCR) using SYBR green chemistry. Total RNA from DB, OCI-LY19, KMH2, L428, DEL and SR-786 cell lines was extracted using TRIzol reagents (Invitrogen, Carlsbad, CA, USA) according to the manufacturer's protocol. cDNA synthesis was conducted using SuperScript III First-Strand Synthesis SuperMix for qRT-PCR (Invitrogen, Carlsbad, CA, USA) from the RNA samples and UHRR (Universal Human Reference RNA). *GAPDH *was used as endogenous control. After first strand synthesis, an equivalent of 16 ng of cDNA were added to three triplicate PCR reactions containing SYBR GreenER qPCR SuperMix for ABI PRISM^® ^(Invitrogen, Carlsbad, CA, USA), 10 μM forward primer and 10 μM reverse primer in a final volume of 10 μl. The SYBR GreenER qPCR SuperMix contains ROX (6-carboxy-X-rhodamine), the passive reference fluorochrome that normalizes for pipetting volume errors. Each reaction was performed in triplicate in 384-well Optical reaction plates that cycled for 2 min at 50°C, 95°C for 10 minutes, followed by 40 cycles of 95°C for 15 seconds, and 60°C for 1 minute on 7900 HT Fast Real-Time PCR System (Applied Biosystems, CA, USA). Fluorescent data were converted into cycle threshold (CT) measurements using the 7900 HT-system sequence detection systems (SDS) software and were exported to Microsoft Excel. The Comparative CT Method calculates relative gene expression using the equation:

Affymetrix GeneChip^® ^Human Genome U133 Plus 2.0 Arrays containing the *CYB5B *probe set 238554_at were used to assess the expression of *CYB5B *in HL cell lines in comparison to microdissected normal germinal centre B cells. Microdissection was preformed on a Zeiss Axioplan 2 microscope equipped with mmi CellCut Plus (Molecular Machines Industries'; Haslett, MI, USA) Technology using 6 μm sections from reactive tonsil tissue. Total RNA was extracted using Allprep extraction kits (Qiagen, ON, Canada) and cRNA preparation was performed following the routine protocol for 2-cycle labeling reactions. 11 μg of labeled cRNA were then hybridized on the array overnight and the arrays were washed, stained and scanned using Affymetrix Fluidics Station 450 and Affymetrix GeneChip Scanner (Affymetrix Inc.; Santa Clara, CA). All microarrays passed homogeneous criteria for testing quality control including present call rates >30% and normalized unscaled standard errors <1.05 (NUSE). Affymetrix gene expression data was pre-processed and normalized by Robust Multichip Average (RMA) in R using Bioconductor [48].

### Flow Cytometric Analysis of Surface Expression of CYB5B on Bone Marrow CD34+ Cells

Bone marrow aspirates were depleted of red blood cells by either treating them with Red Blood Cell Lysis Buffer, or using by Ficoll-Paque Plus density gradient centrifugation (GE Healthcare); each method gave similar results. Treated cells were blocked with normal mouse IgG (Invitrogen) for 20 minutes at 4°C, then washed with 4 volumes PBS+2%FBS. Cells were stained using APC-conjugated anti-CD34 (1/50, StemCell Technologies) and FITC-conjugated R23.1 antibody (1/50), for 45 minutes at 4°C. Cells were again washed with PBS+2%FBS, then resuspended in PBS+1 ug/mL propidium iodide, incubated on ice 10 minutes, then fixed with an equal volume of 2% formaldehyde. Cells were then analyzed on a BD FacsCalibur and using FCS Express software.

### RT-PCR Analysis of *CYB5B *Expression in Normal Peripheral Blood and Bone Marrow Samples Enriched for T and B Cell Subpopulations

Cells were separated using Easy-Sep CD3+ or CD19+ magnetic bead-conjugated antibodies (Stem Cell Inc.). RNA was extracted using Trizol reagent (Invitrogen) and up to 2.5 ug of total RNA was reverse-transcribed (Superscript II, Invitrogen). *GAPDH *primers were used to normalize cDNA loading; when appropriate, cDNAs were diluted using the same buffer composition as used to perform reverse transcription. *GAPDH *and *CYB5B *PCRs were carried out in duplicate tubes under the same cycling conditions and run out on the same gel at the same time (1× TAE, 2% agarose). Bands were quantified using Fluorchem 3.04A (Alpha Innotech Corporation).

PCR conditions: 95°C 1 min, 94°C 15 s-56°C 15 s-72°C 15 s for 32 cycles, 72°C for 5 minutes, hold at 4°C.

Primers (designed using Primer3, http://frodo.wi.mit.edu/) were as follows:

hGAPDH-F: CAGCAAGAGCACAAGAGGAAGAGA

hGAPDH-R: TTGATGGTACATGACAAGGTGCGG (160 bp)

h*CYB5B*-F: CTTCTGATGCCAGAGAAATGC

h*CYB5B*-R: GGATTTGCTTTCCGATGTGTA (197 bp)

### Flow Cytometric Analysis of Surface and Cytoplasmic CYB5B Protein Expression in Cell Lines

Jurkat T-cells (5 × 10^6 ^per tube) were blocked with 1/50 control mouse IgG, then labelled on the cell surface with 1/50 of R23.1-FITC, then washed twice with PBS+2%FBS, stained for non-viable cells using propidium iodide, and fixed in 1% formaldehyde. Alternatively, isotype control IgG-blocked cells were resuspended in Cytofix/Cytoperm solution (BD Biosciences Cytofix/Cytoperm Fixation/Permeabilization Solution Kit), then incubated with 1/100 of R23.1-FITC conjugated mAb. Cells were analyzed using a BD FACSCalibur and FCS Express (version 3, DeNovo Software, Los Angeles).

For blocking experiments, Jurkat T-cells (5 × 10^6 ^per tube) were blocked with 1/50 isotype control mouse IgG_1_, then washed and resuspended in Cytofix/Cytoperm solution as per manufacturer's directions. Permeabilized cells were then resuspended in 25 uL and incubated with isotype control IgG_1 _or unlabelled R23.1 antibody (1/25 of either) for 30 minutes at 4°C. Then, 25 uL containing 0.5 uL R23.1-FITC was added, and cells were incubated an additional 30 minutes at 4°C. Cells were analyzed using a BD FACSCalibur and FCS Express (version 3, DeNovo Software, Los Angeles).

## Competing interests

The authors declare that they have no competing interests.

## Authors' contributions

DM and HC performed the protein array identification of CYB5B, full length expression and Western blotting of recombinant CYB5B. MZ performed immunoprecipitation of CYB5B, subcellular fractionation, Western blotting of 2-D gel transfers to PDVF paper, RT-PCR experiments, and gel purification of *CYB5B *amplicons. JP performed RT-PCR on clinical samples and flow cytometric analysis of bone marrow samples. FF and CS performed the array CGH and gene expression studies. RDG provided HL TMA sections and TMA sector maps. DB and AK were responsible for planning, directing of the research as principal investigators, obtaining research funding, performing literature searches and coordinating the experiments between the DB, AK and DM laboratories and editing the final version of manuscript. All authors contributed equally to the drafting of the manuscript.
